# Higher ultraviolet radiation during early life is associated with lower risk of childhood type 1 diabetes among boys

**DOI:** 10.1038/s41598-021-97469-z

**Published:** 2021-09-20

**Authors:** Kate M. Miller, Prue H. Hart, Robyn M. Lucas, Elizabeth A. Davis, Nicholas H. de Klerk

**Affiliations:** 1 Telethon Kids Institute, Nedlands, 6009 Australia; 2 National Centre for Epidemiology and Population Health, Research School of Population Health, Australian National University, Australian Capital Territory, 0200 Australia; 3 Perth Children’s Hospital, Nedlands, 6009 Australia; 4 University of Western Australia, Crawley, 6009 Australia

**Keywords:** Type 1 diabetes, Epidemiology

## Abstract

Population-level ecological studies show type 1 diabetes incidence is inversely correlated with ambient ultraviolet radiation (UVR) levels. We conducted a nested case–control study using administrative datasets to test this association at the individual level. Cases (*n* = 1819) were children born in Western Australia (WA) from 1980–2014, diagnosed with type 1 diabetes at ≤ 16 years. Controls (*n* = 27,259) were randomly selected from all live births in WA, matched to cases by sex and date of birth. Total ambient erythemal ultraviolet radiation (UVR) doses for each trimester of pregnancy and first year of life were estimated for each individual, using daily NASA satellite data that were date- and geographically-specific. Conditional logistic regression tested the association between UVR dose and case–control status. Type 1 diabetes risk was 42% lower in boys of mothers with third-trimester UVR dose in the highest (compared to the lowest) quartile (p = 0.04). Higher UVR in the first year of life was associated with lower type 1 diabetes risk among boys (p = 0.01). UVR dose was not associated with type 1 diabetes risk in girls. Higher UVR in late pregnancy and early life appear to interact with sex-specific factors to lower type 1 diabetes risk among boys in Western Australia.

## Introduction

The incidence of childhood type 1 diabetes has increased worldwide since the 1950s; in the past decade, incidence has increased at an annual rate of approximately 3%^1^. The rapidity of this increase is too great to be attributed solely to changing genetic risk. This is further supported by worldwide reports of a reduced proportion of children with high-risk HLA genotypes presenting with type 1 diabetes^2^. Seasonal variation in type 1 diabetes incidence (see 3 for review) and higher incidence^4,5^ and prevalence^6^ of type 1 diabetes with higher latitude support an environmental trigger^7^. One candidate environmental factor that varies by both season and latitude is ultraviolet radiation (UVR). Ecological studies testing the correlation between levels of UVR and type 1 diabetes have shown a significant inverse relationship with both incidence^7,8^ and prevalence^6^. In addition, an Australian individual-level study has shown an inverse association between UVR band at the time of diagnosis and type 1 diabetes incidence, that was evident only at low population density^9^.

Over the past decade, UVR exposure has become of increasing interest in relation to the development of autoimmune diseases because of its immunomodulatory actions mediated through production of vitamin D, *cis*-urocanic acid, nitric oxide and stimulation of other pathways^10^. For example, low sun exposure is now widely accepted as a risk factor for multiple sclerosis^11^, and recent studies have shown inverse associations between UVR exposure and risk of eczema^12^ and paediatric inflammatory bowel disease^13^. Previous studies suggest that there may be an optimal exposure window to reduce type 1 diabetes risk, prior to the development of diabetes-related autoantibodies, such as the last few months of gestation^14,15^ or in the first year of life^3,16–18^ at the time of both beta cell proliferation and maturation of the immune system, allowing for persistent protection. No studies have examined long-term individual-level data on UVR and development of type 1 diabetes.

In Western Australia, there is considerable variation in UVR as a result of the extensive latitude range (from 10.5° South to 34.5° South) as well as from the four distinct seasons observed in the Perth metropolitan and outer metropolitan region where the majority of the population resides. This population-based study is the first study to use UVR data that are both date- and geographically-specific to investigate the association between ambient UVR during gestation and the first year of life and type 1 diabetes risk in childhood.

## Results

A total of 29,078 children were included in the study. Of these, 1,819 were children diagnosed with type 1 diabetes (cases) and 27,259 were controls. Selected characteristics are presented in Table 1. The mean age at type 1 diabetes diagnosis was 8.6 years (*SD* 4.1), with a range of 6 months to 16 years. There was a slight male preponderance. Participants resided between 10.5 to 34.5° South latitude and 96.9 to 129.4° East longitude. Maternal age for the total sample ranged between 14 and 49 years (median 28 years). The median gestational age at birth was 39 weeks for both cases and controls (range 22–45 weeks).

**Table 1:** Characteristics of children born in Western Australia between 1980–2014 diagnosed with type 1 diabetes (cases) and population controls.

	Cases	Controls	*p*-value^a^
Total cohort size	1,819	27,259	
Sex of child, female [*n* %]	881 (48.4)	13,200 (48.4)	–
Ethnicity, Caucasian [*n* %]	1,734 (95.3)	24,916 (91.4)	< 0.001
Mother’s age at delivery (years) [mean (*SD*)]	28.6 (5.4)	28.4 (5.2)	0.27
**Parity [*n* %]**			0.84
0	759 (41.7)	11,292 (41.4)	
1	621 (34.1)	9,347 (34.3)	
2	305 (16.8)	4,464 (16.4)	
≥ 3	134 (7.4)	2,156 (7.9)	
Caesarean section [*n* %]	484 (7.2)	1,335 (6.0)	< 0.001
Gestational age at time of birth (months) [mean (*SD*)]	38.7 (1.9)	38.9 (1.9)	< 0.001
Complications during pregnancy [*n* %]	652 (35.8)	9,440 (34.6)	0.29
Birth weight (kg) [mean (*SD*)]	3.4 (0.5)	3.4 (0.6)	0.14
Apgar score	9.1 (0.8)	9.1 (0.7)	0.34
Maternal diabetes [*n* %]	10 (0.6)	49 (0.2)	0.001
Gestational diabetes [*n* %]	27 (1.5)	398 (1.5)	0.93
**Remoteness [*n* %]**			0.02
Metro	1,382 (76.0)	19,991 (73.3)	
Rural	318 (17.5)	5,061 (18.6)	
Remote	119 (6.5)	2,207 (8.1)	
**Social disadvantage (IRSD) [*n* %]**			0.19
0–10%	189 (10.6)	2,631 (10.0)	
11–25%	245 (13.8)	4,022 (15.2)	
26–75%	862 (48.5)	13,070 (49.5)	
76–90%	260 (14.6)	3,476 (13.2)	
91–100%	221 (12.4)	3,222 (12.2)	
**Education and occupation (IEO) [*n* %]**			0.20
0–10%	155 (8.0)	2,118 (8.7)	
11–25%	277 (16.4)	4,335 (15.6)	
26–75%	910 (50.6)	13,364 (51.2)	
76–90%	231 (12.0)	3,177 (13.0)	
91–100%	204 (13.0)	3,427 (11.5)	

*p*-values from independent *t*-tests and chi-squared tests comparing cases and controls.

There was a higher proportion of cases who were Caucasian (95%) compared with controls (91%) (p < 0.001). Cases were more likely to be of younger gestational age at birth and be born via caesarean section than controls. This was evident for both elective and emergency caesarean sections. Cases were also more likely to have a mother with diabetes prior to pregnancy (p = 0.001); however, no differences for maternal gestational diabetes were observed between the two groups (p = 0.93). Further, there were no significant differences observed between cases and controls regarding maternal age, parity, infant birth weight or Apgar score (Table 1).

The mean (*SD*) total UVR dose for the gestation period and first year of life combined was 2408.14 kJ m^−2^ (273.67 kJ m^−2^) and 2426.04 (304.22 kJ m^−2^) for cases and controls respectively. Unadjusted conditional logistic regression showed that total UVR dose was significantly lower among cases than controls during gestation and the first year of life (p < 0.001 and 0.01 respectively). Analysis stratified by sex, showed that this difference was only significant among males and not females. Fractional polynomial analysis indicated no significant departure from linearity of the UVR effects (p ≥ 0.20). As expected, UVR dose and latitude were very highly correlated (r = − 0.94).

No association was observed between total UVR dose in the first or second trimesters and subsequent type 1 diabetes risk in childhood (Table 2). In contrast, total UVR dose in the third trimester was significantly associated with type 1 diabetes risk (adjusted risk ratio (aRR) 0.86 per 100 kJ m^−2^ increase in total UVR dose, 95% CI 0.76, 0.98; p = 0.02). Higher total UVR dose in the first 12 months of life was associated with significantly lower type 1 diabetes risk in later childhood (Table 2). Neither total UVR dose during gestation nor the first year of life was associated with age of onset of type 1 diabetes.

**Table 2:** Adjusted relative risk (95% CI) per 100 kJ m^−2^ increase in total erythemal UVR dose during early life for type 1 diabetes developed by age 16 years in children born in Western Australia between 1980–2014.

	Combined		Boys		Girls	
	Relative risk^a^ (95% CI)	*p* value	Relative risk^a^ (95% CI)	*p* value	Relative risk^a^ (95% CI)	*p* value
First trimester	0.97 (0.88, 1.07)	0.52	0.92 (0.80, 1.06)	0.25	1.01 (0.88, 1.16)	0.87
Second trimester	0.97 (0.89, 1.05)	0.40	0.95 (0.85, 1.06)	0.34	0.98 (0.88, 1.10)	0.76
Third trimester	0.86 (0.76, 0.98)	0.02	0.80 (0.67, 0.95)	0.01	0.95 (0.79, 1.13)	0.57
**Gestation period**^**b**^	0.95 (0.90, 1.00)	0.05	0.92 (0.85, 0.99)	0.02	0.98 (0.91, 1.05)	0.60
0–3 months	0.82 (0.70, 0.96)	0.01	0.71 (0.56, 0.90)	0.004	0.93 (0.75, 1.15)	0.51
3–6 months	0.81 (0.70, 0.95)	0.01	0.75 (0.60, 0.94)	0.01	0.89 (0.71, 1.10)	0.27
6–9 months	0.83 (0.70, 0.97)	0.01	0.76 (0.61, 0.95)	0.02	0.90 (0.72, 1.12)	0.34
9–12 months	0.84 (0.72, 0.96)	0.03	0.76 (0.60, 0.96)	0.02	0.93 (0.75, 1.16)	0.34
First year of life	0.95 (0.91, 0.99)	0.01	0.92 (0.86, 0.98)	0.01	0.97 (0.92, 1.03)	0.37

^a^Models adjusted for ethnicity, maternal age, maternal diabetes, birth weight, parity, IEO, IRSD, caesarean section and gestational age at birth. Gestation models also adjusted for complications during pregnancy.

^b^Trend in gestation trimester (total erythemal UVR dose for first, second and third trimester combined).

**Figure 1:**
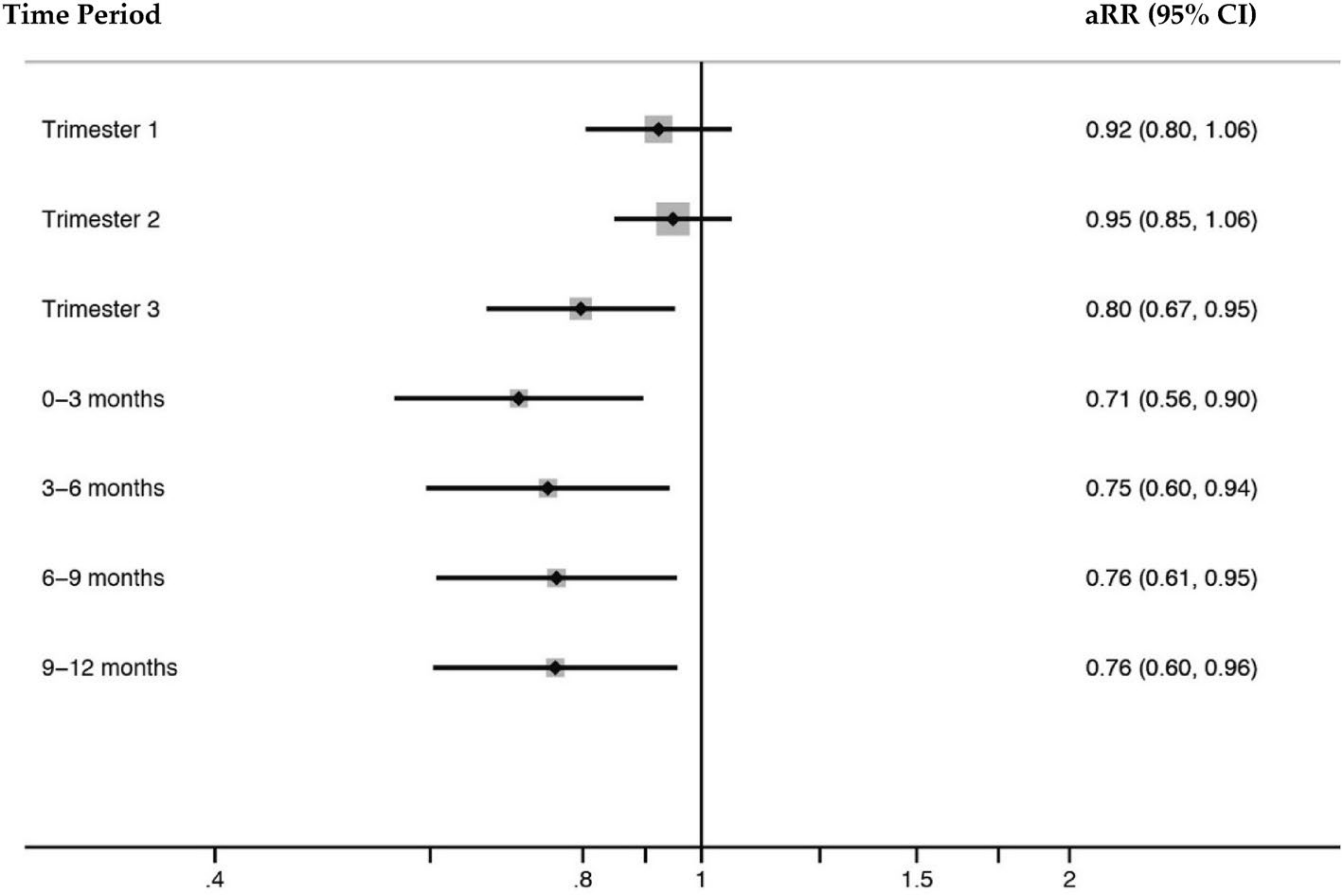
Forest plot of adjusted* relative risk (RR) for type 1 diabetes among boys born in Western Australia per 100 kJ m^−2^ increase in total ambient erythemal UVR dose.

Whilst there was no significant interaction between sex and UVR for any period (p > 0.05 for each of the seven periods), analysis stratified by sex indicated a significant effect of UVR on type 1 diabetes risk in boys but not in girls. Among girls, total UVR dose was not associated with subsequent type 1 diabetes risk in childhood for any of the time periods investigated. However, among mothers of boys, higher total UVR dose in the third trimester was associated with significantly lower type 1 diabetes risk (Fig. 1). When third trimester total UVR dose was considered in quartiles, the risk of type 1 diabetes was 42% lower for the highest quarter compared to the lowest quarter (95% CI [2%, 65%]; p = 0.04). (Table 3; first and second trimester data shown in Table S1 and S2 respectively). Similarly, higher total UVR dose over the first 12 months of life was associated with a significantly lower risk of type 1 diabetes among boys; this effect was most pronounced for the first three months post-birth (Table 2). For every 100 kJ m^−2^ increase in total UVR dose, the relative risk of type 1 diabetes among boys decreased by 29% (total UVR dose ranged from 75.8 kJ m^−2^ to 723.9 kJ m^−2^) (Fig. 1). An interaction analysis indicated that there was no significant variation in effect of UVR by age at diagnosis (gestation p = 0.15; first year of life p = 0.34).

**Table 3:** Adjusted^*^ relative risk (95% CI) for type 1 diabetes developed by age 16 years in children born in Western Australia between 1980–2014 by erythemal UVR during the third trimester.

Quartile^b^	Combined		Boys		Girls	
	Relative risk^a^ (95% CI)	*p* value	Relative risk^a^ (95% CI)	*p* value	Relative risk^a^ (95% CI)	*p* value
Quartile 1	Reference		Reference		Reference	
Quartile 2	0.96 (0.77, 1.19)	0.68	0.99 (0.73, 1.35)	0.98	0.92 (0.67,1.26)	0.60
Quartile 3	0.83 (0.62, 1.12)	0.23	0.84 (0.55, 1.29)	0.44	0.82 (0.53, 1.25)	0.36
Quartile 4	0.67 (0.47, 0.97)	0.03	0.58 (0.35, 0.98)	0.04	0.77 (0.47, 1.27)	0.31

^a^Models adjusted for ethnicity, maternal age, maternal diabetes, birth weight, parity, IEO, IRSD, caesarean section, gestational age at time of birth and complications during pregnancy.

^b^Total sum of UVR is divided into quartiles (IQ1, median IQ3). IQ1 was used as the reference category. Quartiles based on distribution in controls.

## Discussion

In this cohort study, we used linked data from multiple population-based datasets to investigate the association between ambient erythemal UVR dose during gestation (i.e., UVR dose experienced by the mother during pregnancy) and the first year of life and subsequent risk for type 1 diabetes in childhood. Our data show that higher total UVR dose in the third trimester of pregnancy or in the first 12 months of life were associated with reduced risk of developing type 1 diabetes during childhood in boys but not girls. The protective effect of ambient erythemal UVR in the first year of life was most pronounced for the first three months.

Late pregnancy and the first year of life are a critical time for human fetal pancreatic development, which occurs between 24–40 weeks gestation and during the neonatal period. During this time, baseline beta cell mass is determined, and beta cell differentiation occurs^19^. At this time of beta cell development, low sun exposure could negatively impact islet neogenesis, resulting in reduced islet mass and ultimately beta cell deficiency and compromised glucose metabolism^20^. A recent systematic review suggests that exposure to UVR can modulate metabolic function in humans^21^. In mice this type of modulation appears to be through a UVR-induced nitric oxide pathway^22^.

While we cannot rule out the possibility of a type 1 error in our finding of a sex-specific effect, our results are consistent with those of a 2016 Danish case control study which reported an association in boys, but not girls. Notably, that study (cases *n* = 886) found that greater sunshine hours during the third trimester were associated with lower hazard of type 1 diabetes in 5–9-year-old boys only (HR 0.60; p = 0.003 for sunshine hours dichotomised at the median), with the finding not replicated for other age groups^14^. A lack of association across all age groups in the Jacobsen et al. study, and not the present study may be due to differences in methodological approaches. Unlike the Danish study, the present study was able to adjust for a birth cohort effect and sex more precisely (by matching on both date of birth and sex), and season effects by using daily erythemal UVR dose rather than sunshine hours. Furthermore, the present study adjusted for several known and potential confounders. Two other studies that focused on seasonal variation in month of birth also showed an effect in boys but not girls^23,24^, suggesting a sex-specific association between UVR exposure in early life and reduced type 1 diabetes risk in childhood. Thus, sex-related phenomena in early life may enhance the action of UVR-induced molecules acting either directly on the health and survival of beta cells, or indirectly on the immune pathways associated with beta cell destruction and development of type 1 diabetes. There is some evidence from studies on skin cancer development that males have a greater propensity to UVR-induced immune suppression^25^; studies in mice suggest that this may be mediated by testosterone^26^. Testosterone is present in utero and testosterone exposure during gestation begins to differentiate by sex in the second trimester and continues until birth^27^. We propose that testosterone may work with UVR-induced molecules to improve beta cell development, function, and survival, by reducing oxidative stress, insulin resistance and glucose intolerance, thus slowing or preventing beta cell apoptosis in susceptible children. Alternatively (or additionally), if the effect of UVR is via regulating the immune system, there is some evidence of a sex-dependent interaction between the immune system and type 1 diabetes susceptibility. For example, in non-obese diabetic mice interferons acted as both positive or negative modulators of type 1 diabetes risk, dependent on sex^28^. A sex difference in development of islet cell autoantibodies has been previously reported^29^ but, in that study did not translate to a difference according to sex for the development of diabetes, the outcome of interest in our study.

We evaluated the association between type 1 diabetes risk and ambient erythemal UVR dose. Exposure of the skin to UVR is the major source of vitamin D in many locations. A role for vitamin D is possible; beta cells express the vitamin D receptor (*VDR)* and respond to UVR-induced vitamin D by reduced inflammatory and immunogenic cellular activity^10^. Prior studies have shown that higher 25(OH)D levels in the third trimester, but not in the first or second trimesters, were associated with reduced risk of type 1 diabetes in offspring^15,30–32^. Of note, however, is current evidence that suggests maternal intake of vitamin D-only supplements during pregnancy at current recommended levels (400 IU) does not reduce risk of islet autoimmunity (IA) or type 1 diabetes among offspring (see^3^ for review). It is possible that the current recommended dose is not high enough to provide protection or that the benefits of vitamin D supplementation are limited to those for whom insufficiency is corrected or nuanced to particular *VDR* polymorphisms^33^. Importantly, these studies focusing on vitamin D did not show a sex difference in effect, such as was demonstrated in the current study for ambient erythemal UVR dose. Exposure of the skin to UVR results in the synthesis of vitamin D and other immunoregulatory molecules. Vitamin D supplementation provides only vitamin D. The sex-specific effects seen in our study and others may reflect a protective effect of sun exposure that is separate to any effect of vitamin D. Should the benefits of higher UVR exposure be independent of vitamin D as evident for other autoimmune diseases such as multiple sclerosis^34^, eczema^12^ and inflammatory bowel disease^13^, supplementation alone may be insufficient to reduce the risk of type 1 diabetes in children. Further research is needed to determine the relative importance of vitamin D and other immunomodulatory molecules produced in skin, following UVR exposure, as potential regulators of the development and progression of T1D.

Two large-scale Danish studies have reported no association between newborn dried blood spots and subsequent type 1 diabetes risk. Notably, the 25(OH)D levels were low, with 46% and 51% of neonates in the respective studies with levels < 25 nmol L^−1^^35^. A further analysis of data from the Norwegian study by Thorsen et al^32^. reported that higher cord blood 25(OH)D is associated with reduced risk of type 1 diabetes only in children with specific polymorphisms in the *VDR* gene^31^. One case–control study has measured 25(OH)D levels in the first year of life and childhood risk of type 1 diabetes. The study of children with an increased genetic risk of type 1 diabetes (cases = 244, controls = 488), reported lower 25(OH)D levels at 12 months of age among cases compared with controls after adjustment for a range of covariates including month of sample collection, HLA genotype, maternal type 1 diabetes and sex^36^. Studies investigating associated risk of IA have produced mixed results. For example, in the Diabetes Autoimmunity Study in the Young (DAISY) study (including children at increased genetic risk of type 1 diabetes)^37^, serum 25(OH)D levels at 9 months of age (*n* = 128) were not associated with IA in children up to 2 years of age. However, a recent prospective study of children with increased genetic risk of type 1 diabetes (*n* = 1,417), found that vitamin D sufficiency (25(OH)D ≥ 50 nmol L^−1^) in early infancy was associated with a 40% lower risk of IA compared with vitamin D deficiency (25(OH)D < 50 nmol L^−1^)^38^. Further analysis revealed that the association between vitamin D sufficiency and reduced IA risk was only evident in those with specific *VDR* polymorphisms. The DAISY study did not examine interactions between 25(OH)D and *VDR* variants, which may explain the absence of an association in their study, as may the difference in the participant’s ages at the time of sample collection.

A limitation of the current study is that we did not have data on personal UVR exposure for mothers or children, rather we used a proxy of ambient erythemal UVR dose. Sun exposure can vary significantly between individuals as a result of both lifestyle (sun exposure and sun protective behaviours) and cultural factors (clothing and sun protection behaviour including use of sunscreen)^39^ that modify the received UVR dose for any ambient erythemal UVR dose. Nevertheless, ambient UVR (vitamin D-weighted) has been shown to be a significant predictor of serum 25(OH)D level (r^2^ = 0.2)^40^, and vitamin D-weighted UVR and erythemally-weighted UVR are highly correlated, particularly at higher UVR levels such as are typical of Western Australia^41^. While inclusion of individual sun behaviour data may have improved the accuracy of personal UVR dose estimates, there are issues of feasibility for detailed collection over a long time period, as well as considerable challenges with the accuracy of personal sun exposure measurement. Questionnaire data typically use coarse categories that do not account for time of day^13^, while dosimeters measure sun exposure at a particular body location (often the wrist), and even if reliably worn, may not take account of clothing or orientation of the dosimeter to the sun^42^. One additional caveat is that ambient UVR could possibly be a proxy for some other factor that was the true protective agent, such as a dietary factor. The present study was also limited by our inability to identify which children moved out of state during the study period. In addition, we used the postcode recorded in the MNS to infer the mother’s residential location during pregnancy and the child’s location for the first 12 months of life. It is probable that for some, the postcode at birth (and respective UVR measures) did not reflect the location of the mother or child for the whole study period.

This study has several strengths. We used data from a large sample, representative of Western Australian children aged 0–16 years. These data were obtained from linking population-based statewide registries, were of high quality and spanned 35 years^43,44^. Furthermore, the use of ambient erythemal UVR dose, which unlike sunshine hours, also considers the intensity of the UVR and provides a finer scale for variation, is a novel and important feature of the study and allows for international comparisons with future studies. This is the first study to use UVR data that is both date- and geographically-specific, at an individual level, to investigate the association between UVR and type 1 diabetes. Whilst previous Australian studies have reported an association between latitude and/or UVR and type 1 diabetes^4,9^, this is the only study to look at UVR during gestation and the first year of life. Finally, and perhaps most importantly, our ability to accurately calculate gestation and the total UVR dose for critical time periods has provided insight into the optimal window in early life for higher levels of UVR exposure to provide persistent protection for type 1 diabetes throughout childhood.

These data show an association between higher ambient erythemal UVR in the third trimester of pregnancy (maternal UVR dose) and the first year of life, and subsequent decreased risk for type 1 diabetes in childhood among boys. The relationships described in the present study are associations and do not demonstrate a causal effect. Clinical trials would typically be required before behaviour modification could be recommended but such trials may not be feasible. Rather, advice for pregnant women and children to have regular time outdoors, protected from excessive sun exposure according to current Cancer Council guidelines, may be an important public health measure. Further research is required to confirm the proposed interaction between testosterone and UVR-induced molecules in humans. Such knowledge will enhance our understanding about the etiology of type 1 diabetes and the potential of UVR exposure as an intervention to delay, limit or prevent symptomatic disease.

## Methods

We conducted a case–control analysis nested within a cohort study formed using individually linked data from the Western Australian Children’s Diabetes Database (WACDD), the Midwives’ Notification System (MNS) and the Western Australian Deaths Registry. Data linkage was performed by the Western Australia Data Linkage Branch using probabilistic matching (based on key identifiers such as name, address and date of birth)^45^. Daily ambient erythemal UVR data were obtained from NASA's Goddard Space Flight Centre.

Participants were children born in Western Australia between 1 January 1980 and 31 December 2014 who had a record on the MNS, an administrative dataset established from notifications required by Section 335 of the Health (Miscellaneous Provisions) Act 1911, which is complete for > 99% of all births in Western Australia^43^. For the period 1980–2014, there were 867,571 children recorded on the MNS. Of those, 0.34% had incomplete data on key variables. Children were excluded if they were missing data on maternal age (*n* = 16), gestational age (*n* = 1,965), postcode (*n* = 898) or sex (*n* = 55).

Cases were identified using the WACDD, a statewide registry that includes over 99% of Western Australian children newly diagnosed with type 1 diabetes^44^. Cases were restricted to children born in Western Australia with a diagnosis of type 1 diabetes between the ages of 0–16 years. Of the 2,611 children with a record on the WACDD database between 1 January 1987 and 31 December 2014, 1,819 linked with the MNS (i.e., were born in Western Australia). Controls were randomly selected from all live births in Western Australia and matched to cases by sex and date of birth and were still at risk of type 1 diabetes at the case’s date of diagnosis.

Demographic, medical and obstetric information about the mother and information on the labour, delivery, and condition of the infant were obtained from the MNS. Demographic information included postcode and the Australian Bureau of Statistics Socio-Economic Indexes for Areas*,* namely the Index for Relative Social Disadvantage (IRSD) and the Index for Education and Occupation (IEO). Information on the mother included: date of birth, ethnicity, complications during pregnancy, medical conditions, parity, and birth method. Information on the baby included: date of birth, sex, ethnicity, gestational age at birth, height, weight and Apgar score at five minutes after birth. A diagnosis of type 1 diabetes was determined by an endocrinologist using International Society for Pediatric and Adolescent Diabetes guidelines^46^.

Ambient erythemal UVR data were obtained from the Goddard Space Flight Centre Total Ozone Mapping Spectrometer (TOMS) Instrument and Nimbus 7, downloaded from http://iridl.ldeo.columbia.edu/SOURCES/.NASA/.GSFC/.TOMS/, for 1 January 1979 to 31 December 2004, and NASA’s Ozone Monitoring Instrument (OMI), downloaded from https://disc.gsfc.nasa.gov/datasets/OMUVBd_003/summary?keywords=OMI for 1 January 2005 to 31 December 2014. These data have been described previously^47^; in brief, daily dose of ambient erythemal UVR (in J m^−2^) was obtained for every 1° of latitude and 1.25° of longitude from the TOMS satellite and 1° × 1° from the OMI satellite. A UVR working file was created that contained the daily ambient erythemal UVR dose for each day between 1 January 1979 and 31 December 2014 and each latitude and longitude coordinate in Western Australia.

Maternal age was calculated from the mother’s date of birth and the baby’s date of birth. Dichotomous variables were created for caesarean section (yes, including both elective and emergency/no), maternal diabetes (yes, including type 1 and type 2 diabetes/no), gestational diabetes (yes/no), complications during pregnancy (yes/no) and ethnicity (Caucasian/non-Caucasian). The IRSD and IEO scores for each individual were categorised into specified groups (0–10%, 11–25%, 26–75%, 76–90% and 91–100%)^48^, with a higher score indicating greater advantage and higher education. A conception date was calculated for each pregnancy by subtracting the gestational age at birth from the date of birth. First and second trimester dates were then determined from the conception date, adding 12 weeks and 28 weeks, respectively. The date of birth was used for the second trimester date for babies born before 28 weeks and the third trimester date for those born after 28 weeks gestation. For cases, the date of diagnosis (defined as the date of hospital admission) of type 1 diabetes was obtained from the WACDD.

For each individual, UVR data were merged on the latitude and longitude of the residential postcode recorded in the MNS (e.g. 1 March 1990, 32.5° S, 115.5° E). We recorded the daily ambient erythemal UVR dose for the date of birth, the 315 days prior to birth date and the 365 days post-birth. Total UVR dose during pregnancy was calculated by summing the recorded daily ambient erythemal UVR dose over the period from the conception date to the birth date. Total UVR dose for the first trimester was the sum of the daily ambient erythemal UVR dose from conception to day 83; second trimester from day 84 to day 195; and third trimester from day 196 to the date of birth. Total UVR dose for the first year of life was calculated by summing the daily ambient erythemal UVR dose for the 365 days following the date of birth. Total UVR dose for each quarter of the first year was calculated by summing the daily ambient erythemal dose for each day in the relevant quarter. Total UVR dose was considered as a continuous variable and also split by quartiles.

The subjects in this study consist of all children born in Western Australia between 1980 and 2014 and followed up for death or onset of diabetes until the end of 2014. Thus, this is a cohort study with the exposure of interest being satellite-derived ambient erythemal UVR dose. We used nested case–control matching to control for the variation in incidence of type 1 diabetes over calendar time and by age and sex. Cases were classified into strata according to their sex, date of birth and age in days at date of diagnosis. Up to 15 controls were randomly selected for each case, matched from each of these sex-date of birth-age in days combinations using the Stata routine *sttocc*. Conditional logistic regression, with matching strata defined by these group combinations, was then used to estimate effects of ambient erythemal UVR dose on type 1 diabetes risk for each trimester and quarter of the first year of life. Models were adjusted for all available known and potential confounders including ethnicity, maternal age, maternal diabetes, infant weight, parity, IRSD, IEO, caesarean section, gestational age at birth, and complications during pregnancy (gestation models only). A consistent set of covariates was used for each time period within gestation and the first year of life so that the models were comparable to each other. Because a recent publication indicated sex-specific effects of environmental factors on risk of type 1 diabetes, we decided a priori to also conduct analyses stratified by sex^14,15^.

Conditional logical regression was also used (univariately) to compare the distributions of covariates between cases and controls. Simple linear regression was used, where appropriate, to examine relationships between ambient erythemal UVR dose and continuous variables. We tested linearity of the UVR effects using additional fractional polynomial models. As total UVR doses in the different time periods were highly correlated, no models included UVR dose for multiple time periods. Due to the collinearity between latitude and UVR dose, and more importantly, to enable the investigation of the association between UVR dose during different time periods and type 1 diabetes, latitude was excluded from all models. Because of the incidence density sampling of controls, odds ratios from conditional logistic regression estimate incidence rate ratios (RR), which we have referred to as the more generic term relative risk.

To determine whether ambient erythemal UVR dose during gestation or the first year of life was associated with age of onset of type 1 diabetes, we performed linear regression on case data only, using age of onset as the outcome and total UVR dose (for gestation or first year of life) as the exposure, in addition to all covariates and calendar year (because of the changing distribution of diagnosis age over calendar time). The working file for managing the UVR data was constructed in Microsoft Excel, and the main analysis was run in Stata/SE 15.1. The statistical significance level was set a p < 0.05.

This study was approved by the Western Australian Department of Health (2016/05) and the University of Western Australia Human Research Ethics Committee. All methods were carried out in accordance with relevant guidelines and regulations. The WACDD is a consent-based registry. Informed and written consent was obtained, by the treating physician, from newly diagnosed patients and their parent and/or legal guardian at the time of diagnosis, prior to the collection and storage of data. Assent was obtained from children without the capacity to consent.

## Supplementary Information

Supplementary Information 1.

Supplementary Information 2.

## Data Availability

The data that support the findings of this study are available from Western Australian Department of Health, but restrictions apply to the availability of these data, which were used following approval from data custodians and human research ethics for the current study, and so are not publicly available. Data are however available from the authors upon reasonable request and with permission of all relevant data custodians and the Perth Children’s Hospital Human Research Ethics Committee.
