# Supplementary material for: Higher ultraviolet radiation during early life is associated with lower risk of childhood type 1 diabetes among boys
Source: Sci Rep. 2021 Sep 20;11:18597. doi: 10.1038/s41598-021-97469-z (PMC8452739; doi:10.1038/s41598-021-97469-z)
Supplement: Supplementary file 1 — Supplementary Information 1. [file 41598_2021_97469_MOESM1_ESM.pdf]

Table S1: Adjusted relative risk[1] [95% CI] for type 1 diabetes developed by age 16 years in children born in Western Australia between 1980–2014 by ambient erythemal UVR during the first trimester

	**Combined**		**Boys**		**Girls**	
Quartile[2]	Relative Risk[1] (95% CI)	*p* value	Relative Risk[1] (95% CI)	*p* value	Relative Risk[1] (95% CI)	*p* value
Quartile 1	Reference		Reference		Reference	
Quartile 2	0.88 (0.71, 1.09)	0.24	0.83 (0.61, 1.12)	0.21	0.94 (0.69,1.26)	0.66
Quartile 3	1.00 (0.75, 1.33)	1.00	0.98 (0.65, 1.47)	0.92	1.00 (1.66, 1.51)	1.00
Quartile 4	1.03 (0.72, 1.47)	0.86	0.91 (0.55, 1.50)	0.71	1.16 (0.70, 1.94)	0.56

[1]Models adjusted for ethnicity, maternal age, maternal diabetes, birth weight, parity, IEO, IRSD, caesarean section, gestational age at time of birth and complications during pregnancy.
[2]Total sum of UVR is divided into quartiles (IQ1, median IQ3). IQ1 was used as the reference category. Quartiles based on distribution in controls.
