# Supplementary material for: Higher ultraviolet radiation during early life is associated with lower risk of childhood type 1 diabetes among boys
Source: Sci Rep. 2021 Sep 20;11:18597. doi: 10.1038/s41598-021-97469-z (PMC8452739; doi:10.1038/s41598-021-97469-z)
Supplement: Supplementary file 2 — Supplementary Information 2. [file 41598_2021_97469_MOESM2_ESM.pdf]

Table S2: Adjusted* relative risk [95% CI] for type 1 diabetes developed by age 16 years in children born in Western Australia between 1980–2014 by ambient erythemal UVR during the second trimester

	Combined		Boys		Girls	
**Quartile**[2]	Relative Risk[1] (95% CI)	*p* value	Relative Risk[1] (95% CI)	*p* value	Relative Risk[1] (95% CI)	*p* value
Quartile 1	Reference		Reference		Reference	
Quartile 2	1.06 (0.85, 1.32)	0.61	0.99 (0.72, 1.35)	0.94	1.13 (0.83,1.55)	0.43
Quartile 3	1.10 (0.82, 1.47)	0.54	1.00 (0.66, 1.52)	0.99	1.19 (0.78, 1.81)	0.42
Quartile 4	0.94 (0.66, 1.34)	0.72	0.88 (0.53, 1.45)	0.61	1.00 (0.60, 1.65)	0.99

[1]Models adjusted for ethnicity, maternal age, maternal diabetes, birth weight, parity, IEO, IRSD, caesarean section, gestational age at time of birth and complications during pregnancy.
[2]Total sum of UVR is divided into quartiles (IQ1, median IQ3). IQ1 was used as the reference category. Quartiles based on distribution in controls.
